# Anlotinib for previously treated advanced or metastatic esophageal squamous cell carcinoma: A double‐blind randomized phase 2 trial

**DOI:** 10.1002/cam4.3771

**Published:** 2021-02-14

**Authors:** Jing Huang, Juxiang Xiao, Wentao Fang, Ping Lu, Qingxia Fan, Yongqian Shu, Jifeng Feng, Shu Zhang, Yi Ba, Yang Zhao, Ying Liu, Chunmei Bai, Yuxian Bai, Yong Tang, Yan Song, Jie He

**Affiliations:** ^1^ Department of Medical Oncology National Cancer Center/National Clinical Research Center for Cancer/Cancer Hospital, Chinese Academy of Medical Sciences and Peking Union Medical College Beijing China; ^2^ Department of Medical Oncology The First Affiliated Hospital of Xi'an Jiaotong University Xi'an China; ^3^ Department of Thoracic Surgery Shanghai Chest Hospital, Shanghai Jiao Tong University Shanghai China; ^4^ Department of Oncology First Affiliated Hospital of Xinxiang Medical University Xinxiang China; ^5^ Department of Oncology The First Affiliated Hospital of Zhengzhou University Zhengzhou China; ^6^ Department of Medical Oncology The First Affiliated Hospital of Nanjing Medical University Nanjing China; ^7^ Department of Medical Oncology Jiangsu Cancer Hospital, Jiangsu Institute of Cancer Research, Nanjing Medical University Affiliated Cancer Hospital Nanjing China; ^8^ Department of Medical Oncology Shandong Cancer Hospital Jinan China; ^9^ Department of Medical Oncology Tianjin Cancer Hospital Tianjin China; ^10^ Department of Biostatistics School of Public Health Nanjing Medical University Nanjing China; ^11^ Department of Medical Oncology Henan Cancer Hospital Zhengzhou China; ^12^ Department of Medical Oncology Peking Union Medical College Hospital, Chinese Academy of Medical Sciences and Peking Union Medical College Beijing China; ^13^ Department of Medical Oncology Harbin Medical University Cancer Hospital Harbin China; ^14^ Department of Gastroenterology Affiliated Tumor Hospital, Xinjiang Medical University Urumqi China; ^15^ Department of Thoracic Surgical Oncology National Cancer Center/National Clinical Research Center for Cancer/Cancer Hospital, Chinese Academy of Medical Sciences and Peking Union Medical College Beijing China

**Keywords:** adverse events, anlotinib, esophageal squamous cell carcinoma, metastatic, progression‐free survival

## Abstract

**Background:**

Currently, there are no randomized trials on the effect of antiangiogenic therapy in patients with esophageal squamous cell carcinoma (ESCC). The following study investigated the efficacy and safety of anlotinib in patients with advanced ESCC who were previously treated with chemotherapy.

**Methods:**

This randomized, placebo‐controlled, double‐blind phase 2 trial (NCT02649361) was conducted in 13 Chinese hospitals. Eligible patients were adults with histologically confirmed recurrent or metastatic ESCC who were previously treated with chemotherapy, and were randomly assigned (2:1) to receive oral anlotinib 12 mg or placebo on days 1–14 (repeated every 21 days). The primary endpoint was progression‐free survival (PFS).

**Results:**

One hundred and sixty‐five patients were randomly assigned to the anlotinib (*n* = 110) or the placebo (*n* = 55) arm. Median PFS was 3.02 months (95% CI 2.63–3.65) in the anlotinib group and 1.41 months (95% CI 1.38–1.41) in the placebo group (hazard ratio 0.46 [95% CI 0.32–0.66]; *p* < 0.001). The most common treatment‐related adverse events of grade 3 or 4 were hypertension (17 [16%] patients), decreased appetite (6 [6%] patients), and hyponatremia (4 [4%] patients) in the anlotinib group and decreased appetite (2 [4%] patients) in the placebo group. Three (3%) deaths in the anlotinib group were considered as drug related, while there were no treatment‐related deaths in the placebo group.

**Conclusions:**

The use of anlotinib in previously treated, recurrent, or metastatic ESCC patients significantly improved PFS compared with placebo. Our findings suggest that antiangiogenesis might be an important therapeutic target in advanced ESCC.

**Clinical Trials Registration:**

Study of Anlotinib in Patients With Esophageal Squamous Cell Carcinoma (ALTER1102), NCT02649361.

## Graphical abstract

Anti‐angiogenic multi‐target inhibitor anlotinib significantly improved PFS and DCR compared with placebo in this study. Our findings demonstrated that angiogenesis targeting therapy might be an efficient therapeutic target in advanced or metastatic ESCC.

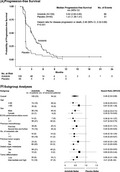

## 1 INTRODUCTION

Esophageal cancer (EC) is the seventh most common malignancy and the sixth leading cause of cancer‐related deaths worldwide.^1^ The global incidence of EC varies across different regions of the world.^2^ There are two main typical histological subtypes of EC worldwide, that is, esophageal squamous cell carcinoma (ESCC) and esophageal adenocarcinoma (EAC). In China, ESCC is the predominant subtype.^2^ Given that the etiology and the molecular characteristics are different; the treatment strategies for ESCC should be developed separately to optimize patient outcomes from EAC.

Over the past decades, advanced or metastatic ESCC has been managed mainly with platinum plus paclitaxel or fluorouracil‐based chemotherapy.^3^, ^4^ However, the overall survival of these patients remains poor.^3^, ^4^ Treatment options in patients with advanced ESCC who progressed on or were intolerant to standard chemotherapy are limited. Docetaxel or paclitaxel has been used as second‐line therapy in EAC because of evidences from the randomized trials, but not in ESCC due to lack of evidence on survival benefit and increased toxicities in patients with ESCC.

Although epidermal growth factor receptor tyrosine kinase inhibitors have been proved to be effective in non‐small cell lung cancer, no definitive efficacy has yet been demonstrated in the treatment of ESCC. The Cancer Oesophagus Gefitinib (COG) trial, the first randomized phase 3 study of systemic targeted therapy in patients with advanced esophageal cancer progressing after chemotherapy, demonstrated that the overall survival did not improve by gefitinib compared to placebo.^5^ More recently, three randomized phase 3 trials reported the promising efficacies of anti‐PD‐1 antibodies in second‐line setting in patients with advanced esophageal cancer as compared with chemotherapy. Phase 3 KEYNOTE‐181 trial showed that overall survival in pembrolizumab group was statistically superior to chemotherapy in advanced esophageal cancer patients with a PD‐L1 combined positive score (CPS) of ≥10.^6^ While, nivolumab and camrelizumab have demonstrated OS benefit in two different randomized trials in patients with ESCC, in which patients were irrespective of PD‐L1 status.^7^, ^8^ It is noteworthy that despite the longer overall survival in anti‐PD‐1 antibody group compared to chemotherapy group observed in these trials, only part of the patients could be benefit from the treatments.

Anlotinib is an oral small‐molecule tyrosine kinase inhibitor (TKI) targeting the vascular endothelial growth factor (VEGF) receptor 1, 2, and 3, fibroblast growth factor (FGF) receptor 1–4, platelet‐derived growth factor (PDGF) receptor ɑ and β, Ret and c‐Kit.^9^ It has also been reported that patients with cervical invasive carcinoma have increased expression of VEGF; while bevacizumab (a VEGF‐targeting monoclonal antibody) is effective against advanced squamous cell carcinoma of the cervix.^10^ The results of these studies suggest a potential benefit of angiogenesis therapy in ESCC. In the present study, we conducted a multicenter, randomized, double‐blind, placebo‐controlled phase 2 trial to evaluate the antitumor activity and safety of anlotinib as second‐line or later‐line therapy in patients with advanced or metastatic ESCC.

## 2 MATERIALS AND METHODS

### 2.1 Study design and participants

This randomized, double‐blind, placebo‐controlled, phase 2 trial was conducted across 13 hospitals in China (NCT02649361). Eligible patients were 18–75 years of age; had histologically confirmed recurrent or metastatic ESCC (stage IV) with at least one measurable lesion diagnosed according to the Response Evaluation Criteria In Solid Tumors (RECIST) version 1.1; had received at least one regimen of platinum‐ or taxane‐based chemotherapy; progressed on or were intolerant to prior chemotherapy; had an Eastern Cooperative Oncology Group (ECOG) performance status score of 0–2; had adequate hematologic, hepatic, renal, and cardiac functions. Key exclusion criteria were: active hemorrhage at the primary lesions during the previous 2 months; primary lesions that were not surgically resectable; prior antiangiogenic therapy that proved to be ineffective; uncontrolled brain metastasis or controlled for less than 3 months. The ethics committee at each study hospital approved the study protocol and all amendments, and the trial was conducted in accordance with Good Clinical Practice guidelines and the principles of the Declaration of Helsinki. Written informed consent was obtained from all patients before enrollment.

### 2.2 Randomization and masking

The patients were randomly assigned (2:1) via centralized randomization system to receive anlotinib or placebo. Randomization was stratified according to metastasis (distant organ metastasis vs. no distant organ metastasis) and tumor differentiation (undifferentiated /poorly differentiated vs. moderately/well differentiated). Patients, medical staff, and investigators were blind to the treatment allocation.

### 2.3 Procedures

Patients received oral anlotinib (12 mg, once per day) or matching placebo in 3‐week cycles. During each therapeutic cycle, the medication was administered for 2 consecutive weeks, followed by 1 week off treatment. Treatment interruptions and dose modifications due to treatment‐related toxicities were allowed. Patients received the assigned study drug until disease progression, unaccepted toxicity, or withdrawal of consent.

Scheduled visits and computed tomography (CT) scans were performed at week 3, 6, and then, every 6 weeks until disease progression. The tumor response was assessed by the investigators and independent central radiologic review based on the RECIST criteria, version 1.1. Safety data were documented during the treatment and within 30 days after the administration of the last drug dose. The investigators graded all adverse events (AEs) according to the National Cancer Institute Common Terminology Criteria for Adverse Events (NCI‐CTCAE) version 4.0. The post‐study data were also collected. All participants were followed up every 2 months for survival status and information of subsequent therapies after completion of the study treatment.

### 2.4 Outcomes

The primary endpoint was investigator‐assessed PFS per RECIST v1.1, which was defined as the time from randomization to disease progression or death from any cause, whichever occurred first. The secondary endpoints were OS (defined as the time from randomization to death from any cause), objective response rate (ORR) (the percentage of patients with a confirmed complete or partial response), disease control rate (DCR) (the percentage of patients with a confirmed complete or partial response, or stable disease), and health‐related quality of life and safety.

### 2.5 Statistical analysis

The trial was designed for comparing anlotinib versus placebo in patients with previous treated recurrent or metastatic ESCC. A total of 144 patients were to be enrolled in 2:1 ratio to observe 114 events, which would provide 80% power to detect a hazard ratio (HR) of 0.56 for PFS of anlotinib group over the placebo with a two‐sided α level of 0.05.

Efficacy analyses were performed in all patients who underwent randomization and were treated with at least one dose of the study drug (full analysis set). Safety analyses were done in all patients who received at least one dose of the study treatment. The PFS and OS were estimated using the Kaplan–Meier method and compared between groups using the log‐rank test. HRs and the associated 95% CIs were calculated using a Cox proportional‐hazards model. The ORR and DCR were compared between groups using Pearson's Chi‐squared or Fisher's exact test, as appropriate. Post hoc subgroup analysis of PFS was performed with a Cox proportional‐hazards model. The SAS 9.2 software (SAS Institute) was used for statistical analyses.

## 3 RESULTS

**FIGURE 1:**
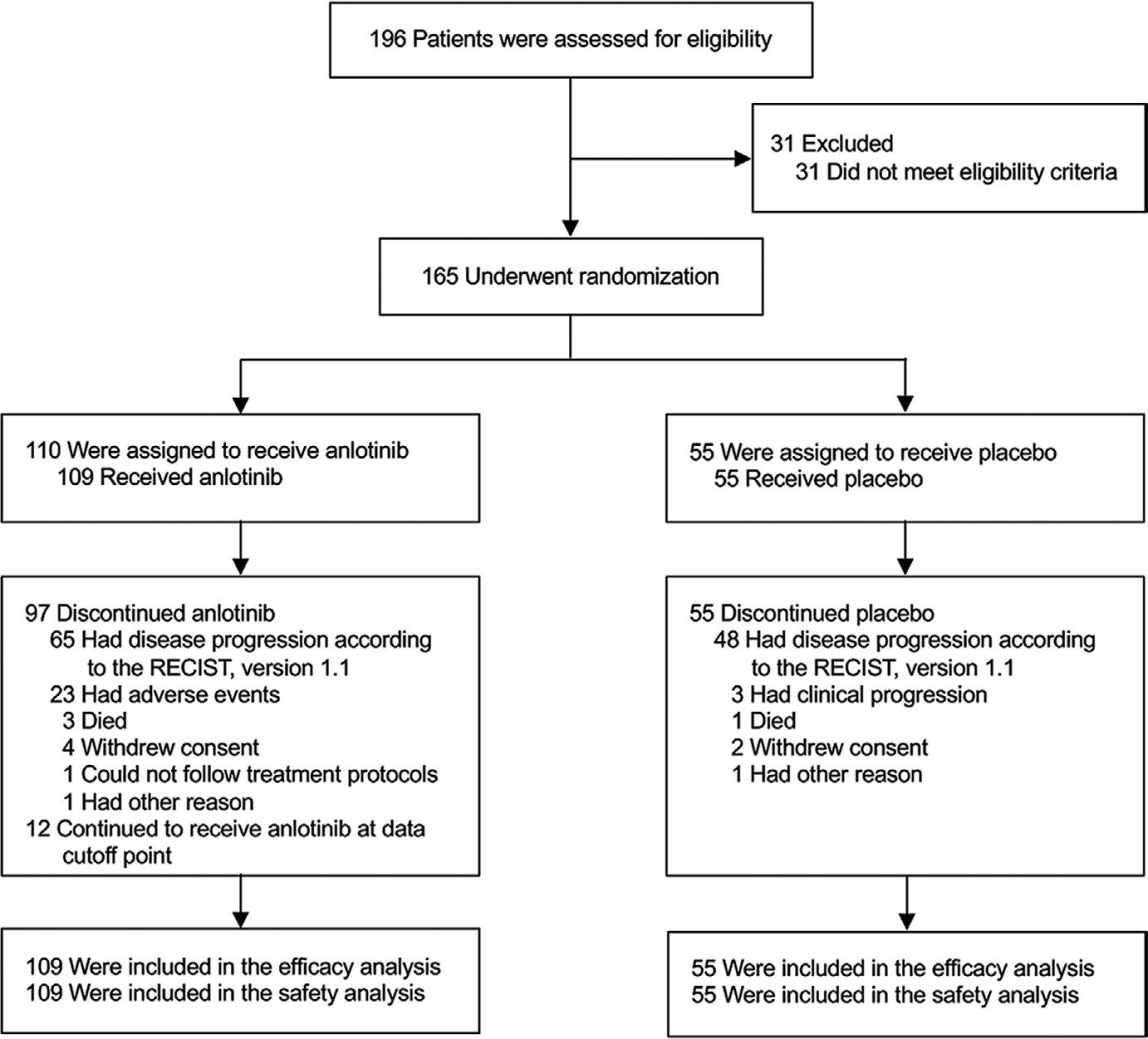
CONSORT Diagram. RECIST, Response Evaluation Criteria in Solid Tumors

Between 6 January 2016 and 22 May 2018, a total of 196 patients were screened for eligibility. A total of 165 patients were randomly assigned (2:1) to either the anlotinib group (*n* = 110) or the placebo group (*n* = 55) (Figure 1). Treatment was initiated in 164 patients, with 109 receiving anlotinib and 55 receiving placebo (full analysis set). Most baseline and disease characteristics of the patients were similar between groups (Table 1). The median age of patients was 62 years in the anlotinib group and 61 years in the placebo group. In both groups, most patients were male and had an ECOG performance status score of 1. In both study groups, 64% of the patients had previously received two or more lines of chemotherapy. The proportion of patients who received previous tumor surgery was 79% in anlotinib group and 60% in placebo group.

**TABLE 1:** Baseline characteristics (full analysis set)

	Anlotinib (*n* = 109)	Placebo (*n* = 55)
Age (years)		
Median	62	61
Range	43–74	45–76
Sex		
Male	86 (79%)	46 (84%)
Female	23 (21%)	9 (16%)
ECOG performance status score		
0	14 (13%)	7 (13%)
1	87 (80%)	43 (78%)
2	8 (7%)	5 (9%)
Number of metastatic sites		
1	16 (15%)	14 (25%)
≥2	93 (85%)	41 (75%)
Distant organ metastasis		
Yes	99 (91%)	49 (89%)
No	10 (9%)	6 (11%)
Tumor differentiation		
Undifferentiated or poorly differentiated	35 (32%)	18 (33%)
Moderately or well differentiated	74 (68%)	37 (67%)
Previous tumor surgery		
Yes	86 (79%)	33 (60%)
No	23 (21%)	22 (40%)
Previous chemotherapy		
One line	39 (36%)	20 (36%)
Two or more lines	70 (64%)	35 (64%)
Previous radiotherapy		
Yes	80 (73%)	42 (76%)
No	29 (27%)	13 (24%)
Comorbidity		
Yes	98 (90%)	50 (91%)
No	11 (10%)	5 (9%)

Data are *n* (%) unless otherwise stated. There were no significant differences between groups at baseline, except for previous tumor surgery (*p* = 0.016).

Abbreviation: ECOG, Eastern Cooperative Oncology Group.

By the data‐cutoff date of 22 July 2018, the median follow‐up was 11.8 months (IQR, 7.2–23.8). Moreover, 97 (88.2%) out of 110 patients in the anlotinib group and all of the patients (100.0%) in the placebo group had permanently discontinued study treatment (Figure 1). At the end of the study, 109 (66.1%) out of 165 patients died, and 137 (83.0%) had disease progression. The median treatment duration was 2.6 months (range, 0.5–20.9) in the anlotinib group and 1.2 months (range, 0.5–10.8) in the placebo group. The proportion of patients who received post‐study treatments was 41% (40 patients) in the anlotinib group and 73% (40 patients) in the placebo group (*p* < 0.001). The post‐study therapies used in the anlotinib and placebo groups included chemotherapy (23 [24%] vs. 30 [55%]), apatinib (a VEGF receptor inhibitor) (10 [10%] vs. 11 [20%]), and programed death‐1 (PD‐1) inhibitors (four [4%] vs. six [11%]).

**FIGURE 2:**
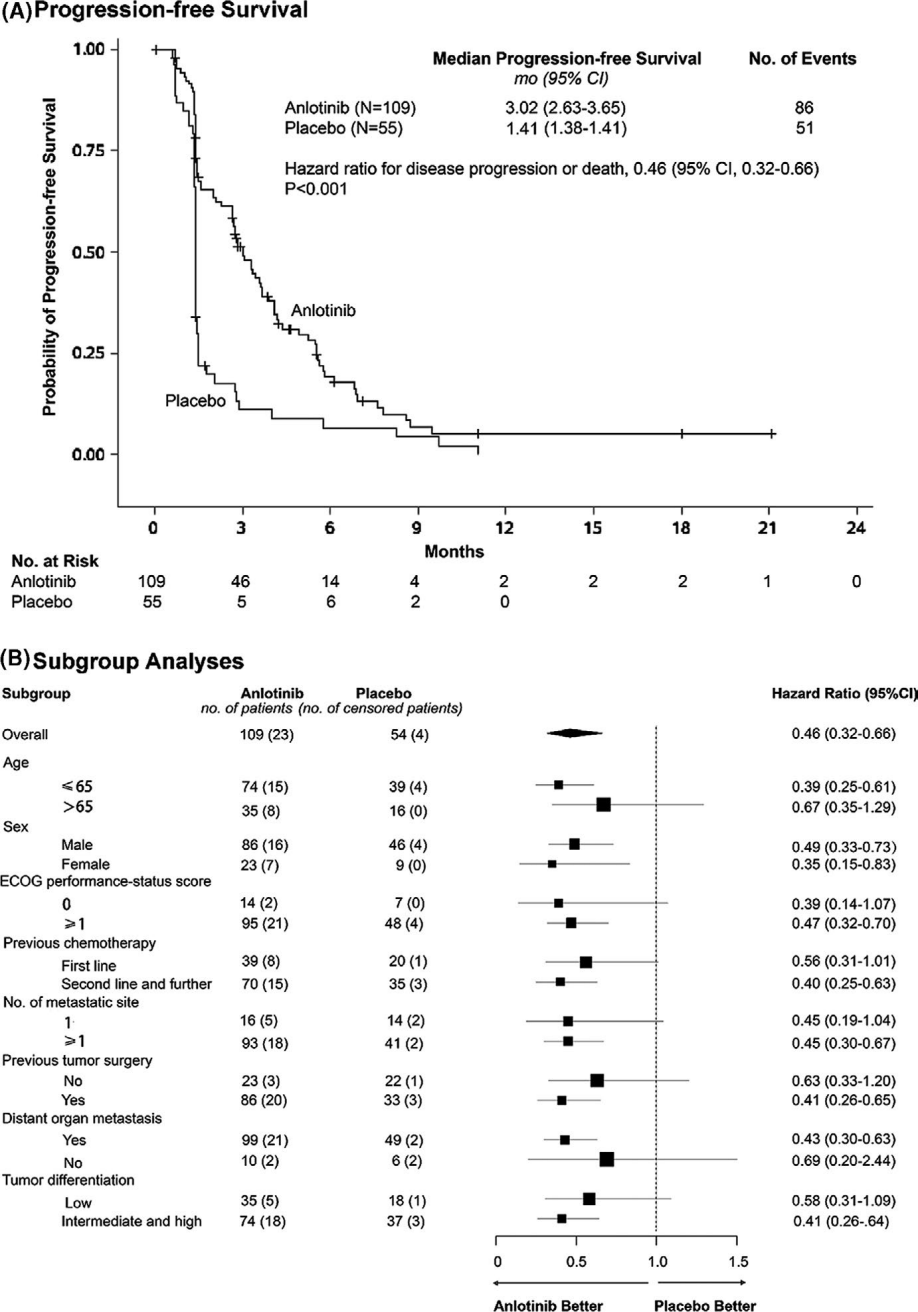
Progression‐free survival. (A) Kaplan–Meier analyses of progression‐free survival (defined as the time from randomization to disease progression or death from any cause, whichever occurred first) in the anlotinib and placebo groups. Cross marks indicate censored observations. (B) Subgroup analyses of progression‐free survival. The analyses of all patients and subgroups were unstratified

The PFS, as assessed by investigator, was longer in patients receiving anlotinib compared to those receiving placebo (HR 0.46 [95% CI 0.32–0.66]; *p* < 0.001; Figure 2A). The investigator‐assessed median PFS was 3.02 months (95% CI 2.63–3.65) in the anlotinib group and 1.41 months (95% CI 1.38–1.41) in the placebo group. The HR was 0.47 (95% CI 0.33–0.69), adjusting for unbalanced history of surgery in baseline characteristics. A PFS benefit with anlotinib was consistently observed across demographic and clinical subgroups (Figure 2B), including patients with disease progression who received two or more lines of chemotherapy (HR 0.40 [95% CI 0.25–0.63]; *p* < 0.001). Consistent with the results provided by investigator, the median PFS was assessed by blinded central review to 2.83 months (95% CI 2.30–3.61) in the anlotinib group compared with 1.41 months (95% CI 1.38–1.41) in the placebo group (HR 0.43 [95% CI 0.30–0.62]; *p* < 0.001).

**FIGURE 3:**
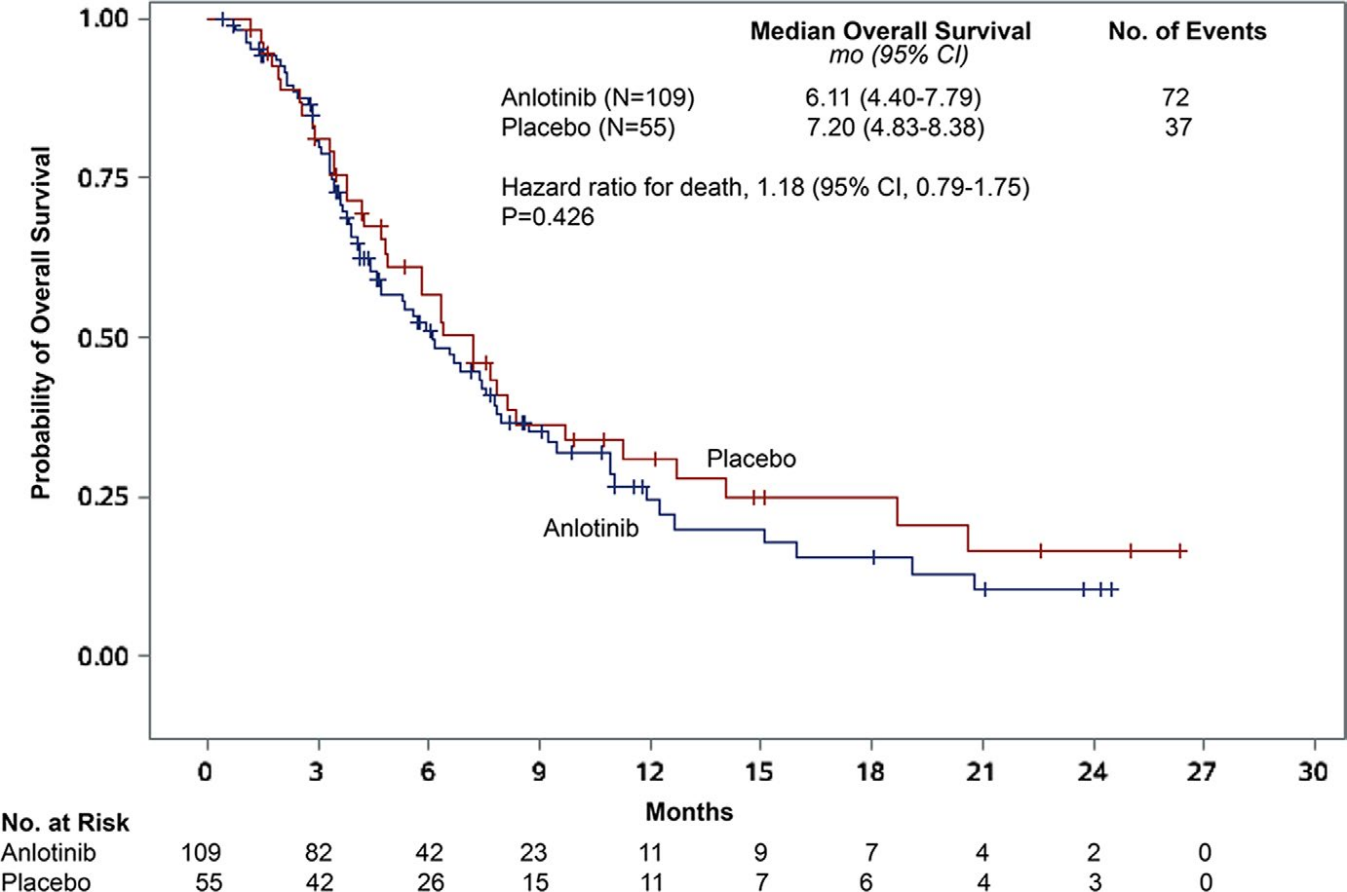
Kaplan–Meier analyses of overall survival. Overall survival was defined as the time from enrollment to death from any cause. Cross marks indicate censored observations

No difference in OS was observed between the two treatment groups (HR 1.18 [95% CI 0.79–1.75]; *p* = 0.426; Figure 3). The median OS was 6.11 months (95% CI 4.40–7.79) in the anlotinib group and 7.20 months (95% CI 4.83–8.38) in the placebo group. After adjusting for unbalanced history of surgery, the HR and log‐rank *p* value were 1.24 (95% CI 0.82–1.88) and 0.2989, respectively.

The ORR was not significantly different between groups (eight [7.3%] of 109 patients vs. two [3.6%] of 55 patients; *p* = 0.498). Notably, two patients in the anlotinib group achieved a complete response, and no disease progression occurred as of the data‐cutoff date (response durations of 18.0+ and 21.1+ months). The median duration of response in all patients who showed a complete or partial response was 5.8 months (95% CI 3.1 to not reach) with anlotinib. The DCR was significantly higher in anlotinib group than that in placebo (70 [64%] out of 109 patients vs. 10 [18%] out of 55 patients, *p* < 0.001), with a higher proportion of patients achieving stable disease in the anlotinib group (62 [57%] out of 109 patients vs. eight [15%] out of 55 patients).

Dose reduction was performed in 27 (25%) out of the 109 patients in the anlotinib group and one (2%) out of the 55 patients in the placebo group. In the anlotinib group, 24 (22%) patients had their dose reduced to 10 mg/d and 3 patients (3%) had their dose reduced to 8 mg/d. Treatment discontinuation due to AEs occurred in 23 (21%) patients in the anlotinib group, and in none of the patients in the placebo group. Treatment‐related AEs of any grade occurred in 102 (94%) out of 109 patients in the anlotinib group and 45 (82%) out of 55 patients in the placebo group (Table 2). The most common treatment‐related AEs of any grade reported in the anlotinib and placebo groups were fatigue (62 [57%] vs. 19 [35%] patients), hypertension (59 [54%] vs. 9 [16%] patients), decreased appetite (47 [43%] vs. 14 [25%] patients), and hypothyroidism (39 [36%] vs. 3 [5%] patients). Grade 3 or 4 treatment‐related AEs occurred in 40 (37%) out of 109 patients in the anlotinib group and six (11%) out of 55 patients in the placebo group. The most common treatment‐related AEs of grade 3 or worse in the anlotinib group were hypertension (17 [16%] patients), decreased appetite (6 [6%] patients), and hyponatremia (4 [4%]). Grade 3 or 4 treatment‐related bleeding was rare, with only two cases (2%) of hemoptysis observed in the anlotinib group and none in the placebo group. Treatment‐related serious AEs were reported in 21 (19%) out of 109 patients in the anlotinib group and one (2%) out of 55 patients in the placebo group. Decreased appetite (three [2%] patients) and hemoptysis (three [2%] patients) were the most common anlotinib‐related serious AEs. Treatment‐related grade 5 AEs occurred in three patients in the anlotinib group. These treatment‐related grade 5 AEs were all bleeding events. One patient with pulmonary metastases achieved partial response, and developed grade 5 hemoptysis 9 months after initiation of the study treatment. The second patient had grade 5 bronchial hemorrhage 2 months after treatment with anlotinib. The target lesions of this patient were all in the lung and the overall response were stable disease. The third patient developed anastomotic stricture after surgical resection before enrollment, and had grade 5 upper gastrointestinal bleeding when undergoing endoscopic dilatation 2 months after treatment with anlotinib. There were no grade 5 treatment‐related AEs in the placebo group.

**TABLE 2:** Treatment‐related adverse events

	Anlotinib (*n* = 109)			Placebo (*n* = 55)		
	Any grade	Grade 3	Grade 4	Any grade	Grade 3	Grade 4
Any adverse event	102 (94%)	37 (34%)	3 (3%)	45 (82%)	6 (11%)	0
Fatigue	62 (57%)	2 (2%)	0	19 (35%)	1 (2%)	0
Hypertension	59 (54%)	17 (16%)	0	9 (16%)	0	0
Decreased appetite	47 (43%)	6 (6%)	0	14 (25%)	2 (4%)	0
Hypothyroidism	39 (36%)	1 (<1%)	0	3 (5%)	0	0
Palmar‐plantar erythrodysesthesia	29 (27%)	2 (2%)	0	0	0	0
Proteinuria	28 (26%)	1 (<1%)	0	7 (13%)	0	0
Thyroid stimulating hormone elevation	26 (24%)	1 (<1%)	0	1 (2%)	0	0
Weight loss	21 (19%)	3 (3%)	0	3 (5%)	0	0
Diarrhea	19 (17%)	0	0	4 (7%)	0	0
Leukopenia	18 (17%)	0	0	2 (4%)	0	0
Aspartate aminotransferase elevation	18 (17%)	2 (2%)	0	2 (4%)	0	0
Prolonged QT interval on the ECG	17 (16%)	2 (2%)	0	6 (11%)	0	0
Hypercholesterolemia	16 (15%)	0	0	4 (7%)	0	0
Hypertriglyceridemia	15 (14%)	0	0	4 (7%)	0	0
Gamma‐glutamyltransferase elevation	15 (14%)	3 (3%)	0	3 (5%)	1 (2%)	0
Alanine aminotransferase elevation	15 (14%)	1 (<1%)	0	6 (11%)	0	0
Dysphonia	15 (14%)	0	0	5 (9%)	0	0
Increased low density lipoprotein	14 (13%)	1 (<1%)	0	2 (4%)	0	0
Lymphocytopenia	14 (13%)	2 (2%)	0	3 (5%)	0	0
Oropharyngeal pain	13 (12%)	0	1 (<1%)	2 (4%)	0	0
Abdominal pain	13 (12%)	0	0	1 (2%)	0	0
Globulin urine present	12 (11%)	0	0	2 (4%)	0	0
Neutrophil count decreased	12 (11%)	0	0	1 (2%)	1 (2%)	0
Rash	11 (10%)	0	0	2 (4%)	0	0
Nausea	11 (10%)	0	0	3 (5%)	1 (2%)	0
Hyperglycemia	5 (5%)	0	0	6 (11%)	0	0
Insomnia	1 (<1%)	0	0	1 (2%)	1 (2%)	0

Data are *n* (%). Treatment‐related adverse events occurring at any grade in more than 10% of patients or grade 3 or worse in more than 1% of patients in the safety population are shown in this table. Events are listed in descending order of frequency (any grade) in the anlotinib group. Treatment‐related grade 5 adverse events occurred in three (3%) patients were hemoptysis, pulmonary hemorrhage, and upper gastrointestinal hemorrhage in the anlotinib group and none in the placebo group.

## 4 DISCUSSION

To the best of our knowledge, this is the first randomized controlled trial to evaluate the efficacy of an angiogenesis inhibitor in recurrent or metastatic ESCC. Our data revealed that anlotinib might significantly prolong the PFS by 1.6 months (3.02 vs. 1.41 months) with a HR of 0.46 in patients with previously treated recurrent or metastatic ESCC. The primary endpoint had reached. In addition, the safety profile of anlotinib was acceptable. Thus, our findings suggest that antiangiogenesis might be an important therapeutic target in advanced or metastatic ESCC.

There is limited data on the effectiveness of targeted therapies for metastatic ESCC in a second‐line or further setting. Previously, COG trial evaluated gefitinib for advanced chemotherapy‐refractory esophageal cancer with histologically both adenocarcinoma and squamous cell carcinoma. It was revealed that the OS was not improved by gefitinib versus placebo (3.73 vs. 3.67 months) in genetically unselected ESCC patients; while the median PFS was only marginally prolonged (1.57 vs. 1.17 months).^5^ In a phase 2 trial of icotinib, which is another EGFR TKI, the PFS and OS in previously treated metastatic ESCC patients with EGFR overexpression or EGFR gene amplification were 52 days and 153 days, respectively.^11^ As to immune checkpoint inhibitors, the treatments with PD‐1 inhibitor lead to encouraging response rates of around 20% and long durable responses.^6^, ^7^, ^8^ The present study showed that anlotinib might lead to a median PFS of 3.0 months in patients with chemotherapy‐refractory metastatic ESCC, with a clinically meaningful 1.6 months longer than that in patients given placebo. Notably, our data indicated that the median PFS in the placebo group was 1.4 months, compared to the 1.17 months for the placebo group in the COG trial, showing the aggressiveness of advanced ESCC. Interestingly, recent studies have revealed a complex relationship between VEGF signaling and anticancer immunity, and clinical trials have suggested a synergistic effect between anti‐PD‐L1 and anti‐VEGF therapies.^12^ Future studies might explore the possible benefit of dual therapy with anlotinib and an immune checkpoint inhibitor for the treatment of advanced ESCC.

The objective response rate and the disease control rate for anlotinib in our study were 7% and 64%, respectively. By comparison, the COG trial reported an objective response rate of 4% and a disease control rate of 24% in unselected patients with esophageal cancer treated with gefitinib.^5^ In addition, the icotinib phase 2 trial reported an objective response rate of 17% and a disease control rate of 46% in patients with EGFR‐overexpressing ESCC.^11^ The improvement in PFS observed with anlotinib in our study is mainly due to an increase in the proportion of patients with stable disease, rather than an increased proportion response rate observed for anlotinib was modest, the duration response was of responders. Although the remarkable, with a median duration of 5.8 months (95% CI, 3.1 to not reached).

Two main reasons could potentially explain similar OS between the anlotinib group and the placebo group. First of all, the ratio of patients with metastatic sites of ≥2 was higher in anlotinib (85%) than that in placebo (75%). The proportion of patients with liver, lung, and pleural metastasis in anlotinib group was 33%, 67%, and 5%, respectively. In placebo group, the proportion of each metastatic organ was 22%, 55%, and 2%, respectively. Apparently, patients in anlotinib group suffered worse situation with more metastatic organs than that in placebo, and that might be related to the shorter survival duration. Second, it was notable that a higher proportion of patients in the placebo group received post‐study treatment as chemotherapy (55% vs. 24%), apatinib (another inhibitor of the VEGF receptor; 20% vs. 10%), and PD‐1 blockade (11% vs. 4%). These findings may imply that, in addition to anlotinib, the availability of other new treatment options prolonged OS in those patients in present study.

In the present study, all these AEs were observed, and no unexpected AEs or new safety signals were identified. The incidence of grade 3 or worse AEs was more frequent in the anlotinib group than that in the placebo group (39% vs. 11%). Previous studies have reported that the most common AEs associated with anlotinib are hypertension, hypothyroidism, diarrhea, dyslipidemia, proteinuria, and hand‐foot syndrome.^13^, ^14^, ^15^, ^16^ Bleeding events were a major concern for antiangiogenic therapy, especially in patients with SCC. In previous trials of bevacizumab, grade 3 and grade 5 bleeding occurred in 10% and 5% of patients with squamous head and neck cancer,^17^ whereas grade 3 or worse and grade 5 bleeding occurred in 5% and 0% of patients with cervical cancer.^10^ In order to avoid bleeding, it might be reasonable to exclude the ESCC patients who did not received radical tumor surgery or had ulcers from clinical trials of antiangiogenic therapy. Other antiangiogenic treatment‐related AEs of interest included hypertension and proteinuria. The incidence rates of grade 3 or 4 hypertension and proteinuria in our study were comparable with the rates reported in previous phase 3 trials of bevacizumab in advanced cancers (hypertension 16% and proteinuria 1% in the present trial vs. hypertension 4–17.4% and proteinuria 0.6–8.5% in previously phase 3 trials). ^10^, ^18^, ^19^

The limitations of this study include a relatively small sample size and only Chinese patients as subjects. In addition, PFS, but not OS, was the primary endpoint of the present study, although patients were previously treated. Moreover, quite a few participants received new approved drug which might bring more bias in OS evaluation than PFS.

In conclusion, treatment with anlotinib monotherapy resulted in longer PFS and a better DCR compared to placebo in patients with previously treated recurrent or metastatic ESCC. However, the OS did not show benefit. This could be due to the confounding effect of subsequently therapy since more patients in the control group received subsequent therapy compared with patients in the treatment group.

## CONFLICT OF INTEREST

JH: advisory board for Merck and Jiangsu Hengrui. All the remaining authors have declared no conflicts of interest.

## AUTHOR CONTRIBUTIONS

JHu and JHe conceived and designed the study. JHu, JHe, JX, WF, PL, QF, YS, JF, SZ, YB, YL, CB, YB, YT and YS conducted the trial and collected data. YZ performed statistical analysis. JHu and JHe interpreted the data and wrote the manuscript. All authors approved the final version of the manuscript. The corresponding author had full access to all the data in the study, and all authors participated in the decision to submit for publication.

## Data Availability

All data generated or analyzed during this study are included in this published article.
